# Multi‐Stimuli Responsive Magneto‐Coacervate Droplets for Selective Molecular Enrichment and Programmable Manipulation

**DOI:** 10.1002/advs.76398

**Published:** 2026-07-11

**Authors:** Kailang Liu, Haocheng Ran, Haohui Ou, Peiying Chen, Cheng Qi, Tiantian Kong, Zhou Liu

**Affiliations:** ^1^ College of Chemistry and Environmental Engineering Shenzhen University Shenzhen Guangdong China; ^2^ College of Mechatronics and Control Engineering Shenzhen University Shenzhen Guangdong China; ^3^ Department of Biomedical Engineering, School of Medicine Shenzhen University Shenzhen Guangdong China; ^4^ Department of Urology, Shenzhen Institute of Translational Medicine, The First Affiliated Hospital of Shenzhen University Shenzhen Second People's Hospital Shenzhen Guangdong China

**Keywords:** coacervate, condensate, droplet, magnetic actuation

## Abstract

Coacervate droplets formed via liquid–liquid phase separation offer unique opportunities as microreactors and delivery vehicles due to their ability to selectively concentrate biomolecules and support biochemical reactions. However, their passive nature severely restricts precise spatial and temporal control, posing barriers to practical implementation. Here, we report magneto‐coacervate droplets constructed from gelatin, poly(diallyldimethylammonium chloride) (PDDA), and superparamagnetic Fe_3_O_4_@SiO_2_ nanoparticles. These magneto‐coacervate droplets exhibit reversible sol–gel transitions controlled by temperature and pH, tunable surface charge properties for electrostatically driven molecular enrichment, and precise three‐dimensional manipulation under external magnetic fields. These magneto‐coacervate droplets can serve as multifunctional platforms, greatly increasing the practical use of phase‐separated microreactors in environmental remediation, biochemical processing, and targeted biomedical interventions. Demonstrated applications include efficient capture and recycling of microplastic pollutants, magnetically enhanced catalytic enzyme cascades with reaction rates 2–3 times higher than those of conventional methods, and targeted vascular embolization through controlled in situ gelation.

## 1 Introduction

Coacervate droplets, formed by liquid–liquid phase separation (LLPS) of oppositely charged or otherwise interactive macromolecules, have a membraneless, fluid interior and a diffuse interface. These features enable rapid, controllable molecular exchange while maintaining high local concentrations of retained biomolecules [1, 2, 3, 4, 5, 6]. This unique environment not only protects cargo for targeted release but also supports efficient catalytic or biochemical reactions [7, 8, 9, 10], making coacervate droplets both effective delivery vehicles and versatile microreactors in drug formulation, biocatalysis, and tissue engineering [11, 12, 13, 14]. Additionally, many coacervate droplets respond reversibly to environmental cues such as pH [15, 16], ionic strength [17], or temperature [18, 19]. This responsiveness allows selective dissolution or gelation [20, 21], expanding their utility in applications that require the regulation of dynamic processes.

Despite their potential in various applications, most coacervate droplets are passive in solution, relying on random diffusion or bulk flow to reach target sites [22, 23, 24]. In scenarios requiring precise spatiotemporal control, such as targeted drug delivery [25, 26], patterned in situ biocatalysis [27], or multi‐step reaction networks [28, 29], this lack of maneuverability becomes a critical limitation [30, 31]. Traditional droplet manipulation techniques, like electrowetting, are incompatible with coacervate droplets because the dielectric constants of droplets and their diluted phases are nearly identical, preventing effective actuation [32, 33, 34]. Similarly, optothermal approaches [35] depend on tightly focused laser heating and suffer shallow light penetration, limiting manipulation to near‐surface regions. Consequently, there is a pressing need for an alternative external field‐based strategy that can penetrate deeply into materials and provide robust, three‐dimensional control independent of dielectric properties and optical constraints.

Magnetically guided control allows for remote, contact‐free navigation in complex environments, enhancing the functionality of coacervate droplets [36, 37, 38, 39]. However, integrating paramagnetic materials into fully aqueous coacervate droplets remains challenging because coacervate droplets and their diluted surroundings share similar chemical properties, density, and dielectric constants [40, 41]. In contrast, water–oil or water–air droplets benefit from distinct physicochemical differences that simplify particle confinement [42, 43]. Attempts to directly mix iron oxide nanoparticles into coacervates often fail. Even when iron oxide nanoparticles are coated with poly(acrylic acid) to improve water dispersibility, they precipitate upon contact with oppositely charged species [44]. An alternative approach involved forming a water‐immiscible magnetic colloidal coacervate using core–shell iron oxide nanoparticles coated with polyethylene glycol (PEG) chains containing disulfide bonds and anthryl groups. These nanoparticles self‐assembled via π–π stacking during a prolonged dialysis process. While this colloidal material demonstrated navigational capability [45], it lacked the fluid interior and diffusive interface typical of coacervates; such locomotion did not enhance the capacity of coacervates for selective molecular diffusion. As a result, its locomotion did not enhance the coacervates' ability for selective molecular diffusion, dynamic cargo exchange, and interior reactions. This highlights the need for a magnetically responsive coacervate that maintains a fully liquid nature, enabling efficient cargo handling and supporting biochemical or catalytic processes under external field manipulation.

In this work, we introduce magneto‐coacervate droplets, an actively tunable droplet system integrating gelatin, poly(diallyldimethylammonium) (PDDA), and Fe_3_O_4_@SiO_2_ nanoparticles. These droplets spontaneously form in neutral aqueous solutions through electrostatic interactions between gelatin and PDDA, with nanoparticles robustly anchored within the coacervate matrix via hydrogen bonding. The resulting magneto‐coacervate droplets maintain fluid‐like interiors and exhibit strong magnetic responsiveness, enabling precise three‐dimensional manipulation under external magnetic fields. Furthermore, they display dynamic, reversible responses to environmental stimuli: dissolving completely in acidic conditions (pH < 4) and undergoing temperature‐induced sol–gel transitions while retaining fluid characteristics at physiological conditions. Utilizing a programmable electromagnetic coil array, we achieve controlled droplet operations such as translation, rotation, deformation, splitting, and fusion. Leveraging these capabilities, we demonstrate the droplets' diverse functionality as dynamic microreactors to significantly enhance enzyme‐cascade reactions, as magnetically steerable microplastic scavengers for environmental remediation, and as therapeutic agents for targeted vascular embolization via precise delivery and localized gelation. Collectively, magneto‐coacervate droplets substantially advance the versatility and applicability of conventional coacervate systems, offering new opportunities for active and precise interventions in environmental protection, catalytic bioprocessing, and biomedical therapies.

## 2 Results

**FIGURE 1:**
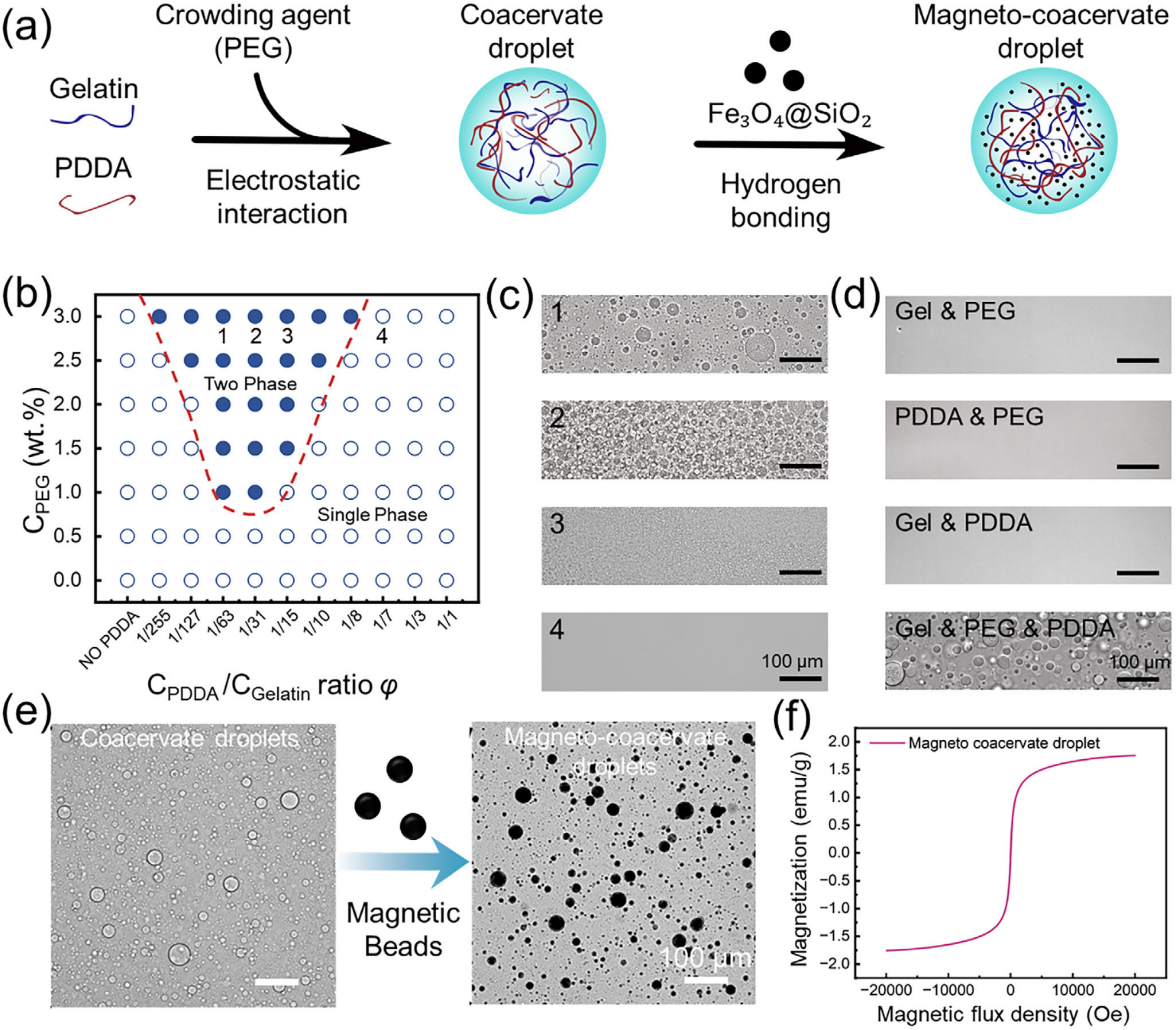
Preparation and characterization of magneto‐coacervate droplets. (a) Schematic illustrating the preparation of magneto‐coacervate droplets. Gelatin and PDDA spontaneously form coacervate droplets through electrostatic interactions in the presence of polyethylene glycol (PEG), a macromolecular crowding agent. Magnetic Fe_3_O_4_@SiO_2_ nanoparticles are subsequently incorporated via hydrogen bonding, yielding magnetically responsive droplets (magneto‐coacervate droplets). (b) Phase diagram depicting conditions for LLPS as a function of PDDA‐to‐gelatin mass ratio (*φ*) and PEG concentration (C_PEG_). The total polymer concentration (gelatin, PDDA) was fixed at 4 wt%. Filled blue circles represent conditions resulting in LLPS, while open circles indicate single‐phase (homogeneous) conditions. (c) Optical micrographs corresponding to numbered points (1–4) in panel (b), demonstrating the significant effect of PDDA‐to‐gelatin ratio (*φ*) on the morphology and yield of coacervate droplets under fixed PEG concentration. Scale bar: 100 µm. (d) Control experiments illustrating that all three components (gelatin, PDDA, and PEG) are required for successful droplet formation. Only the complete ternary mixture produces well‐defined coacervate droplets (*φ* = 1:31, total polymer concentration = 4 wt%, C_PEG_ = 3 wt%). Scale bar: 100 µm. (e) Bright‐field microscopy images comparing gelatin/PDDA coacervate droplets before and after loading Fe_3_O_4_@SiO_2_ nanoparticles, confirming their homogeneous and stable incorporation within magneto‐coacervate droplets. Scale bar: 100 µm. (f) Magnetic hysteresis loop of magneto‐coacervate droplets, measured from −20 to 20 kOe, demonstrating their superparamagnetic properties suitable for precise magnetic manipulation.

To construct magneto‐coacervate droplets with tunable phase behavior and enhanced functionality, we developed a three‐component system consisting of gelatin, poly(diallyldimethylammonium) (PDDA), and polyethylene glycol (PEG) as a macromolecular crowding agent, as illustrated in Figure 1a. Gelatin and PDDA spontaneously form coacervate droplets through electrostatic interactions, undergoing LLPS to yield dense droplets suitable for encapsulation and subsequent functionalization. We systematically explored this coacervation process by mapping a comprehensive phase diagram that illustrates droplet formation as a function of PDDA‐to‐gelatin mass ratio (*φ*) and PEG concentration (C_PEG_), keeping the total polymer concentration constant at 4 wt% (Figure 1b). Coacervation occurred only within a defined window of *φ* (approximately between 1:255 and 1:8), while outside this range, phase separation was inhibited. Furthermore, PEG concentration significantly influenced droplet formation, as coacervates formed exclusively above a critical threshold (≥0.5 wt%). Representative optical micrographs at selected conditions (points 1–4 in Figure 1b,c) clearly demonstrate that the PDDA‐to‐gelatin ratio regulates both droplet morphology and yield. Control experiments further verified that coacervation strictly requires the coexistence of gelatin, PDDA, and PEG, highlighting that the LLPS process is driven by synergistic effects from both electrostatic interactions and macromolecular crowding (Figure 1d).

To confer robust magnetic responsiveness, Fe_3_O_4_@SiO_2_ nanoparticles were successfully integrated into the droplets, forming magneto‐coacervate droplets. The nanoparticles were stabilized by coating with a silica shell via the Stöber method. This protective SiO_2_ coating effectively inhibits oxidation of Fe_3_O_4_, preserving its high saturation magnetization, while simultaneously enhancing biocompatibility and providing abundant surface hydroxyl groups for strong hydrogen bonding with gelatin's amino and carboxyl groups [46]. TEM images of the Fe_3_O_4_@SiO_2_ magnetic nanoparticles revealed a well‐defined core–shell structure, with particle diameters ranging from 10 to 30 nm (Figure S1). EDS elemental mapping confirmed the successful formation of a SiO_2_ shell layer on the Fe_3_O_4_ core surface. Subsequent SEM‐EDS characterization of the magneto‐coacervate microdroplets loaded with Fe_3_O_4_@SiO_2_ nanoparticles demonstrated effective nanoparticle incorporation within the microdroplet matrix (Figure S2). Additionally, FTIR spectroscopy provided direct evidence of these hydroxyl groups; specifically, the strong peak at 1124.1 cm^−^^1^ (Si─O─Si) in Fe_3_O_4_@SiO_2_ nanoparticles (blue curve) confirms the successful SiO_2_ shell encapsulation, while the peak at 618.3 cm^−^^1^ (Fe─O) verifies the Fe_3_O_4_ core structure. Moreover, the characteristic peaks observed at 1627.7 cm^−^^1^ (CONH) and 1219.2 cm^−^^1^ (N^+^(CH_3_)_3_) in the magneto‐coacervate (red curve) spectrum confirm the presence of gelatin and PDDA components, respectively. Importantly, the variation at 3285.4 cm^−^^1^ (O─H) indicates the synergistic interaction between surface hydroxyl groups of SiO_2_ and the hydrophilic structures within the magneto‐coacervate. Bright‐field microscopy images confirmed the homogeneous and stable incorporation of nanoparticles, clearly visualized as uniformly distributed dark spots within magneto‐coacervate droplets (Figure 1e). To validate the stability of Fe_3_O_4_@SiO_2_ nanoparticles encapsulated within coacervate droplets, the magneto‐coacervate droplets (*φ* = 1:31, C_PEG_ = 3 wt%) containing 10 wt% Fe_3_O_4_@SiO_2_ nanoparticles were maintained at 40°C for 12 h and monitored via bright‐field microscopy. As illustrated in Figure S4, the nanoparticles exhibited uniform dispersion within the droplets, with no detectable diffusion into the external medium, indicating no observable nanoparticle release. These results demonstrate that the silica coating and hydrogen‐bonding network effectively ensure stable nanoparticle encapsulation, fulfilling the integrity requirements for magnetically responsive coacervate droplets in magnetic manipulation applications. Magnetic characterization revealed that the resulting droplets exhibit superparamagnetic behavior, with negligible residual magnetization after removing the magnetic field, ensuring reversible and precise magnetic manipulation in aqueous environments (Figure 1f). Collectively, these findings demonstrate the successful development of magneto‐coacervate droplets with tunable physicochemical characteristics and magnetic responsiveness, paving the way for their versatile applications in controlled catalysis, environmental remediation, and targeted therapeutic interventions.

**FIGURE 2:**
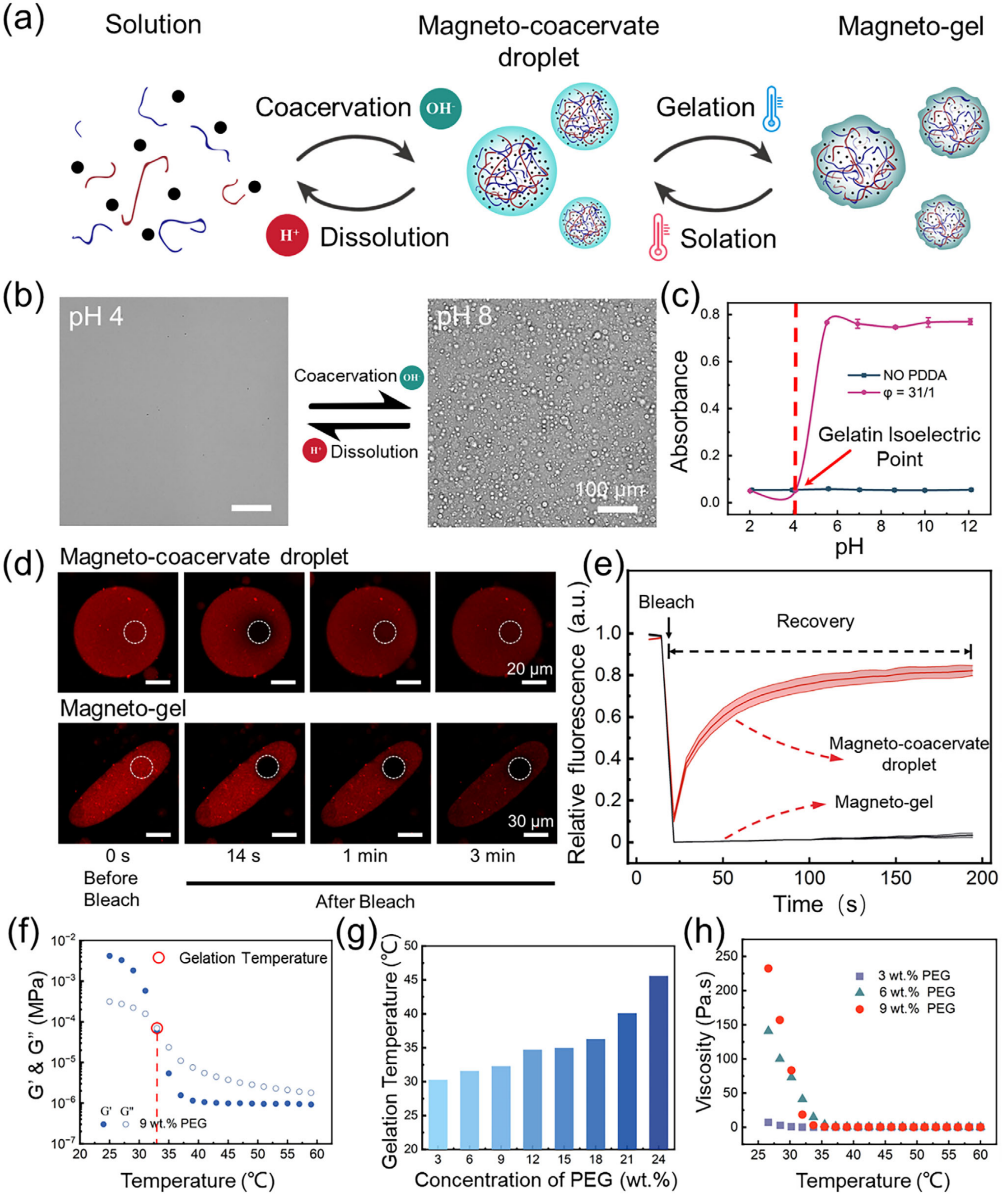
Characterization of pH‐ and temperature‐responsive behaviors and rheological properties of magneto‐coacervate droplets. (a) Schematic illustrating the dynamic responsiveness of magneto‐coacervate droplets composed of gelatin, PDDA, and Fe_3_O_4_@SiO_2_ nanoparticles to pH and temperature stimuli. (b) Bright‐field microscopy images clearly demonstrate reversible pH‐responsive transitions: droplets completely dissolve at acidic pH (pH 4) and reassemble under alkaline conditions (pH 8). Scale bar: 100 µm. (c) Turbidity measurements (absorbance vs. pH) reveal a sharp transition at the gelatin isoelectric point (pI ≈ 4), underscoring PDDA's critical role in mediating droplet formation, as confirmed by control experiments performed in the absence of PDDA. (d) Fluorescence microscopy images highlighting pronounced differences in molecular mobility between the liquid‐like (bottom row) and gelled (top row) states of magneto‐coacervate droplets at different temperatures, revealed by fluorescence recovery after photobleaching (FRAP). (e) Quantitative FRAP recovery curves confirm rapid fluorescence recovery in magneto‐coacervate droplets, indicative of liquid‐like dynamics, contrasted by significantly slower recovery rates in the gelled state, clearly identifying the temperature‐induced sol–gel transition. (f) Rheological analysis of magneto‐coacervate droplets showing temperature‐dependent storage modulus (*G*') and loss modulus (G''). The gelation temperature is precisely defined at the crossover point of *G*' and *Gs*''. (g) Influence of PEG concentration (3–9 wt%) on the gelation temperature, demonstrating tunable thermoresponsive properties of magneto‐coacervate droplets. (h) Temperature‐dependent viscosity profiles of magneto‐coacervate droplets, exhibiting a dramatic viscosity reduction above the gelation temperature, further confirm their pronounced thermosensitive behavior and clear reversible sol–gel transitions.

Apart from magnetic responsiveness, the magneto‐coacervate droplets exhibited distinct and reversible pH‐ and temperature‐responsive phase behaviors, highlighting their multifunctional adaptability, as illustrated in Figure 2a. Owing to the abundance of ionizable groups in gelatin and PDDA, magneto‐coacervate droplets underwent dynamic phase transitions regulated by changes in solution pH. Specifically, the droplets were stable under alkaline conditions (e.g., pH 8) but completely dissolved into a homogeneous, clear solution under acidic conditions (pH < 4) (Figure 2b). Turbidity measurements confirmed that this reversible dissolution and reassembly precisely occurred around the gelatin isoelectric point (∼pH 4), reflecting protonation‐induced charge modulation that disrupts electrostatic interactions between gelatin and PDDA at low pH. Crucially, control experiments conducted without PDDA showed no detectable phase separation across the tested pH range, underscoring PDDA's indispensable role in promoting coacervate droplet formation (Figure 2c). The potential applications of the pH‐responsive characteristics of magneto‐coacervate droplets include magnetic‐targeted drug delivery systems, where microspheres remain stable under physiological pH conditions but selectively dissolve in acidic microenvironments to achieve precise therapeutic release. Furthermore, in environmental or bioprocessing scenarios, this reversible behavior enables the capture of contaminants under neutral pH conditions and their controlled release under acidic conditions. Furthermore, the stability of magneto‐coacervate droplets was notably influenced by ionic strength, as shown in Figure S5. With increasing NaCl concentration (0–200 mM), droplet turbidity progressively decreased due to enhanced electrostatic shielding, which weakens interactions between gelatin and PDDA. At higher ionic strengths, droplet structures were significantly disrupted, ultimately leading to complete dissolution. This salt‐induced dissolution behavior highlights the electrostatic nature of coacervate formation and offers an additional means of externally tuning droplet stability and responsiveness.

The constructed magneto‐coacervate droplets also display prominent temperature‐responsive behavior characterized by reversible sol–gel transitions. Fluorescence recovery after photobleaching (FRAP) experiments clearly illustrated significant differences in molecular mobility between droplets (liquid‐like state at higher temperatures) and gels (solid‐like state at lower temperatures). Rapid fluorescence recovery in the droplet state indicated dynamic molecular diffusion, whereas substantially slower fluorescence recovery in the gel state reflected restricted mobility due to network solidification (Figure 2d,e). Rheological measurements further quantified this temperature‐dependent transition by identifying the precise gelation temperature, defined as the intersection of storage modulus (*G*′) and loss modulus (*G*″) during temperature sweeps (Figure 2f). Importantly, adjusting the PEG concentration allowed precise control over the gelation temperature, with higher PEG concentrations systematically raising this critical transition point (Figure 2g). Additionally, temperature‐dependent viscosity measurements demonstrated a dramatic viscosity reduction at temperatures above the gelation point, confirming a pronounced sol–gel transition (Figure 2h). To evaluate magnetic responsiveness upon gelation, hysteresis loops were measured and compared in droplet and gel states (Figure S6). Both exhibited soft magnetic behavior (Hc < 100 Oe), but the gel showed lower saturation magnetization (∼32% decrease) and a broader hysteresis loop. This arises from the restricted movement of magnetic nanoparticles within the gelatin polymer network, thereby limiting the alignment of magnetic dipoles [47].

**FIGURE 3:**
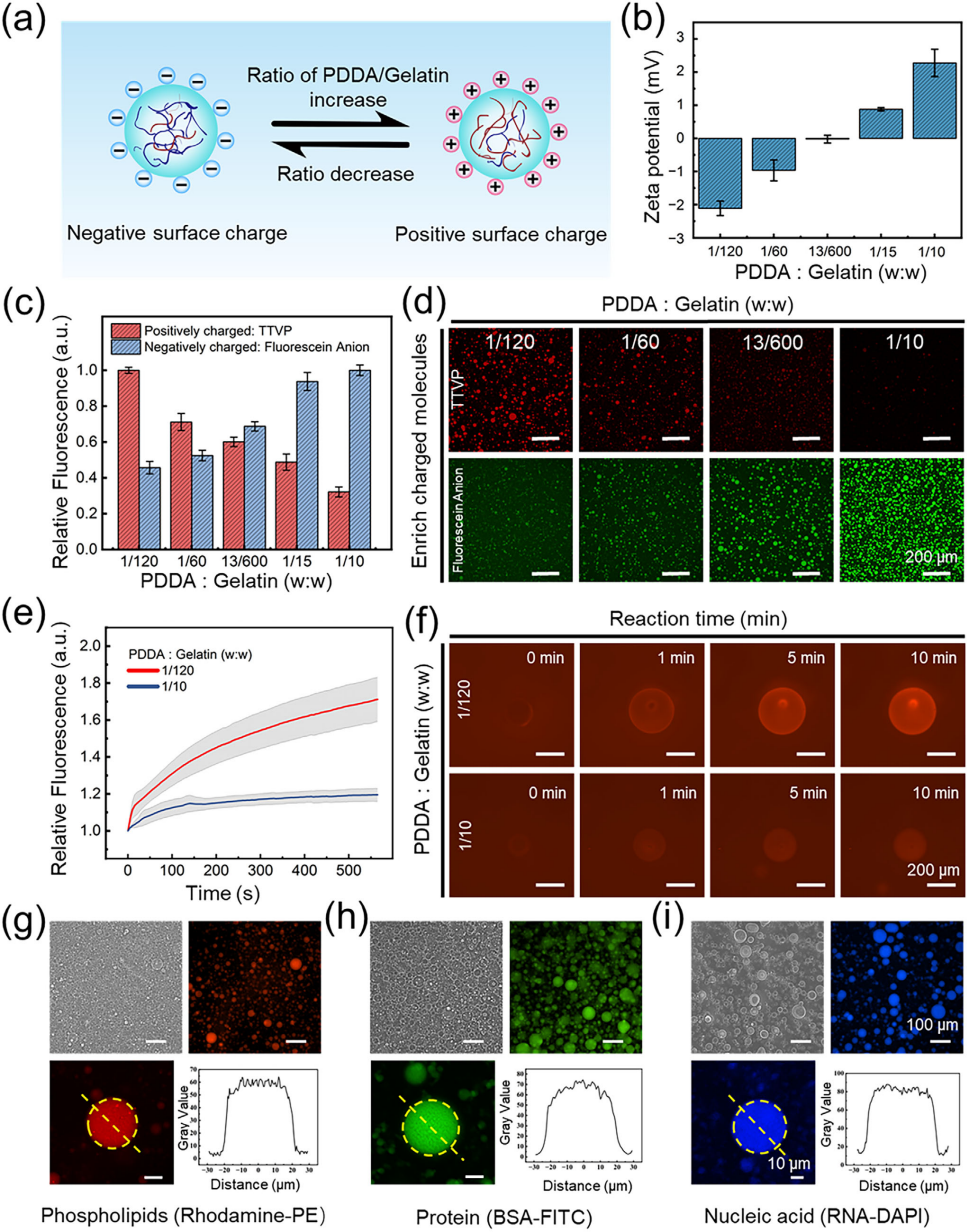
Surface charge modulation and molecular enrichment characterization of magneto‐coacervate droplets. (a) Schematic illustrating the modulation of droplet surface charges by adjusting the PDDA‐to‐gelatin mass ratio (w/w). (b) Zeta potential measurements (mean ± SD, *n* = 3) of magneto‐coacervate droplets at varying PDDA‐to‐gelatin ratios (1:120 to 1:10). (c) Quantitative analysis of selective molecular uptake demonstrating charge‐based enrichment: negatively charged droplets (low PDDA ratio) strongly enrich positively charged TTVP dye (red), while positively charged droplets (high PDDA ratio) preferentially accumulate negatively charged fluorescein anion (green). Bars represent mean ± SD (*n* = 3). (d) Fluorescence microscopy images confirming the selective enrichment trend. The top row images show decreasing TTVP fluorescence as droplet charge transitions to positive; the bottom row images illustrate a sharp increase in fluorescein fluorescence beyond the charge inversion point. Scale bars = 200 µm. (e, f) Kinetics of TTVP enrichment into magneto‐coacervate droplets at low (1:120, red curves/images) and high (1:10, blue curves/images) PDDA‐to‐gelatin ratios. Droplets with lower ratios demonstrate rapid and extensive TTVP enrichment, while droplets with higher ratios exhibit reduced uptake efficiency. Shaded regions represent standard deviation; scale bars = 200 µm. (g–i) Broad‐spectrum biomolecular capture by magneto‐coacervate droplets. Bright‐field (left) and corresponding fluorescence microscopy images (right) confirm efficient encapsulation of diverse bioactive molecules: (g) phospholipids (Rhod‐PE, red), (h) proteins (BSA‐FITC, green), and (i) nucleic acids (RNA‐DAPI, blue). Lower panels depict enlarged droplets with fluorescence intensity line‐scans clearly indicating internal biomolecule localization (yellow dashed outlines; scale bars = 10 µm), demonstrating the droplets' versatility as selective biomolecular carriers.

The magneto‐coacervate droplets developed from gelatin, PDDA, and Fe_3_O_4_@SiO_2_ nanoparticles exhibit tunable surface charge properties, allowing selective and programmable molecular enrichment based on electrostatic interactions (Figure 3a). By systematically varying the PDDA‐to‐gelatin mass ratio (from 1:120 to 1:10), the surface zeta potential of the magneto‐coacervate droplets transitioned from negative to positive values, as shown in Figure 3b. This controllable surface charge modulation provided an effective mechanism for selective molecular recruitment. To investigate this property, we tested two charged fluorescent model compounds: positively charged TTVP and negatively charged fluorescein anion. Quantitative fluorescence analysis showed that negatively charged magneto‐coacervate droplets (lower PDDA ratio, e.g., 1:120) exhibited strong enrichment of positively charged TTVP molecules. Conversely, as the PDDA ratio increased and the droplet surface charge became increasingly positive, TTVP enrichment diminished while the uptake of negatively charged fluorescein anions markedly increased, as shown in Figure 3c. Fluorescence microscopy images confirmed this selective distribution of charged molecules within droplets across different PDDA‐to‐gelatin ratios, demonstrating clear switching in selectivity based on droplet charge polarity, as shown in Figure 3d. We further evaluated the dynamic enrichment process by tracking the kinetics of TTVP uptake into magneto‐coacervate droplets over time. Droplets with a low PDDA ratio (1:120) showed rapid, pronounced, and sustained accumulation of TTVP fluorescence, indicative of stronger electrostatic interactions and higher enrichment efficiency, as shown in Figure 3e,f. In contrast, droplets with a high PDDA ratio (1:10) displayed slower kinetics and markedly reduced fluorescence enrichment due to electrostatic repulsion between positively charged droplets and positively charged TTVP. Moreover, magneto‐coacervate droplets demonstrated broad applicability in selectively capturing various biomolecular cargos, including phospholipids (rhodamine‐labeled liposomes), proteins (BSA‐FITC), and nucleic acids (mRNA stained with DAPI), as shown in Figure 3g–i. Bright‐field and corresponding fluorescence microscopy images revealed efficient encapsulation and homogeneous distribution of these biomolecules within the droplets, further confirmed by fluorescence intensity line scans. These results highlight the potential of magneto‐coacervate droplets as versatile and programmable biomolecular carriers capable of selective enrichment and encapsulation of diverse bioactive molecules for multifunctional applications.

**FIGURE 4:**
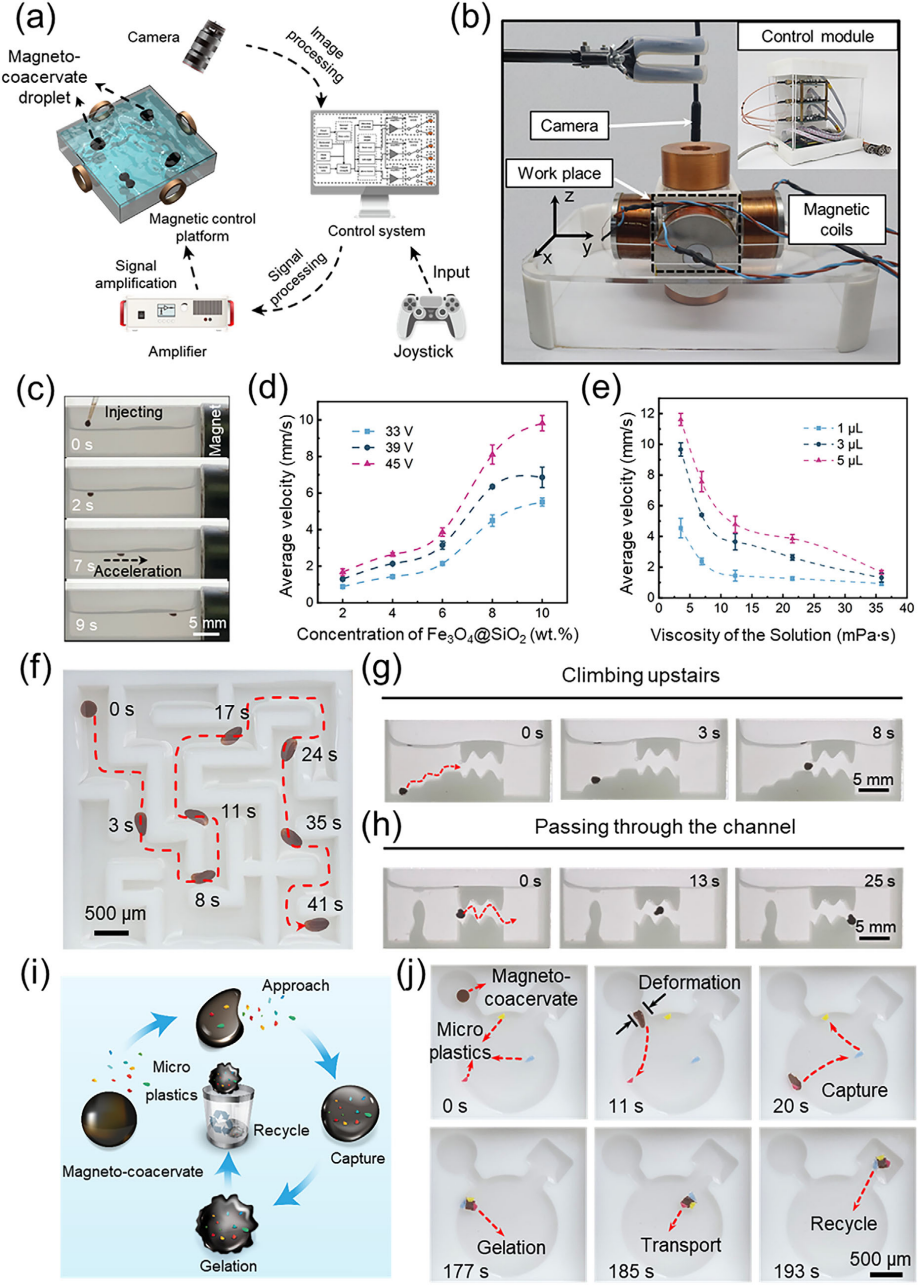
Programmable magnetic manipulation of magneto‐coacervate droplets and their application in microplastic capture and recycling. (a) Schematic of the electromagnetic manipulation platform comprising a multi‐coil array and real‐time optical feedback, enabling precise three‐dimensional magnetic control (transportation, rotation, deformation) of magneto‐coacervate droplets via programmable gradient magnetic fields. (b) Photograph of the constructed electromagnetic coil platform, highlighting the coil configuration and modular design optimized for versatile droplet manipulation. (c) Sequential images illustrating the rapid injection and magnetic‐field‐induced acceleration of magneto‐coacervate droplets, demonstrating robust magnetophoretic responsiveness. (d) Quantitative analysis showing that droplet propulsion velocity increases systematically with higher Fe_3_O_4_@SiO_2_ nanoparticle loading (2–10 wt%) and applied coil voltage (33–45 V), measured at fixed droplet volume (3 µL) and medium viscosity (6.90 mPa·s). (e) Effect of carrier fluid viscosity and droplet volume on magneto‐coacervate droplet velocity, highlighting the influence of environmental conditions on magnetically induced propulsion. (f) Time‐lapse images depicting magneto‐coacervate droplets precisely navigating through a complex maze via programmed magnetic fields, demonstrating centimeter‐scale path‐following capability with high spatial precision. (g) Magneto‐coacervate droplets performing vertical “stair‐climbing” motions against gravity, illustrating robust magnetic maneuverability. (h) Magneto‐coacervate droplets adaptively deforming to pass through narrow and complex microchannel structures, showcasing their viscoelastic nature and shape adaptability under magnetic actuation. (i) Schematic illustrating the complete cycle of microplastic capture, gelation‐based immobilization, magnetic transport, and controlled release by magneto‐coacervate droplets. (j) Experimental demonstration of magnetically guided microplastic capture by droplets, followed by gelation to immobilize captured plastics, subsequent transport to a recovery zone, and reheating to release plastics for recycling. Error bars represent standard deviation (*n* = 3 independent samples). The images in panels c, g, and h were captured from a side view, whereas panels f and j were obtained using a top View.

To translate the physicochemical versatility of magneto‐coacervate droplets into practical functionalities, we established a programmable magnetic manipulation platform comprising a six‐coil, tri‐orthogonal electromagnetic coil array integrated with real‐time optical feedback, as illustrated in Figure 4a [47]. droplet locomotion, deformation, splitting, and fusion in aqueous environments. The spatial distribution and strength of the magnetic fields generated by the coil system were quantitatively characterized, showing an exponential decay of magnetic flux density with increasing axial distance and a linear relationship between magnetic flux density and applied voltage, enabling accurate and predictable droplet manipulation (Figure S7). To provide quantitative insight into droplet propulsion under magnetic fields, we systematically examined the effects of Fe_3_O_4_@SiO_2_ nanoparticle loading and applied coil voltage on droplet velocity, as shown in Figure 4c. The results indicated a monotonic increase in droplet velocity with higher nanoparticle concentrations and coil voltages, achieving a maximum velocity of approximately 9.8 mm/s at a particle loading of 10 wt% and coil voltage of 45 V, owing to the stronger magnetic forces generated at higher particle concentrations (Figure 4d). Conversely, increasing the viscosity of the surrounding fluid by adjusting PEG concentration (6–38 mPa·s, Table S1) predictably decreased droplet velocity due to increased viscous drag; however, larger droplets (5 µL) partially compensated for this increased resistance, maintaining velocities above 5 mm/s even under highly viscous conditions (Figure 4e). Together, these data serve as critical design guidelines for tailoring magneto‐coacervate droplet size, composition, and magnetic actuation parameters to match specific environmental requirements.

Employing predefined magnetic field sequences, a 3 µL magneto‐coacervate droplet successfully navigated a complex labyrinth, completing multiple sharp turns and avoiding dead ends within 41 s, demonstrating centimeter‐scale precision navigation capabilities (Figure 4f). Additionally, droplets effectively performed vertical “stair‐climbing” movements against gravity, ascending a total vertical height of 2.5 mm within 8 s, indicating robust capability to overcome hydrostatic resistance (Figure 4g). Moreover, droplets passed smoothly through narrow microchannels (1.2 mm gaps), adaptively deforming without fragmentation, highlighting their unique viscoelasticity and shape adaptability under magnetically induced interfacial stresses (Figure 4h). Further demonstrating their adaptive viscoelastic properties, magneto‐coacervate droplets exhibited pronounced deformation and controlled splitting under a bidirectional magnetic field (50 mT), where droplets progressively elongated, formed fluid bridges, underwent necking, and ultimately split due to capillary‐driven rupture when magnetic tensile stresses exceeded critical thresholds (Figure S8).

Capitalizing on their magnetic steerability and temperature‐induced sol–gel transitions, magneto‐coacervate droplets were further employed as active, reusable scavengers for environmental microplastic remediation (Figure 4i). Under magnetic guidance, droplets actively approached and enveloped dispersed polystyrene microplastics (50–100 µm), after which cooling below their gelation temperature immobilized the captured plastics securely within their gelled matrix (Figure 4j, 0–20 s). Subsequently, the gelled droplet‐microplastic composites were magnetically transported to a designated recovery zone for downstream recycling processes (177–193 s). It is noteworthy that the interaction between coacervate droplets and microplastics predominantly involves adsorption forces. Consequently, even after reheating and returning to the liquid state, the microplastics remain adsorbed on the droplet surfaces. To achieve controlled release of adsorbed polystyrene microplastics, additional stimuli (e.g., adjusting the solution pH to acidic conditions) are required to disrupt the droplet structure, thereby releasing the encapsulated microplastics into the surrounding medium. This complete cycle of magnetic capture, gelation‐based immobilization, controlled transport, and reversible cargo release exemplifies the practical utility and multifunctional capabilities of magneto‐coacervate droplets, demonstrating their great promise for responsive, sustainable environmental cleanup applications.

**FIGURE 5:**
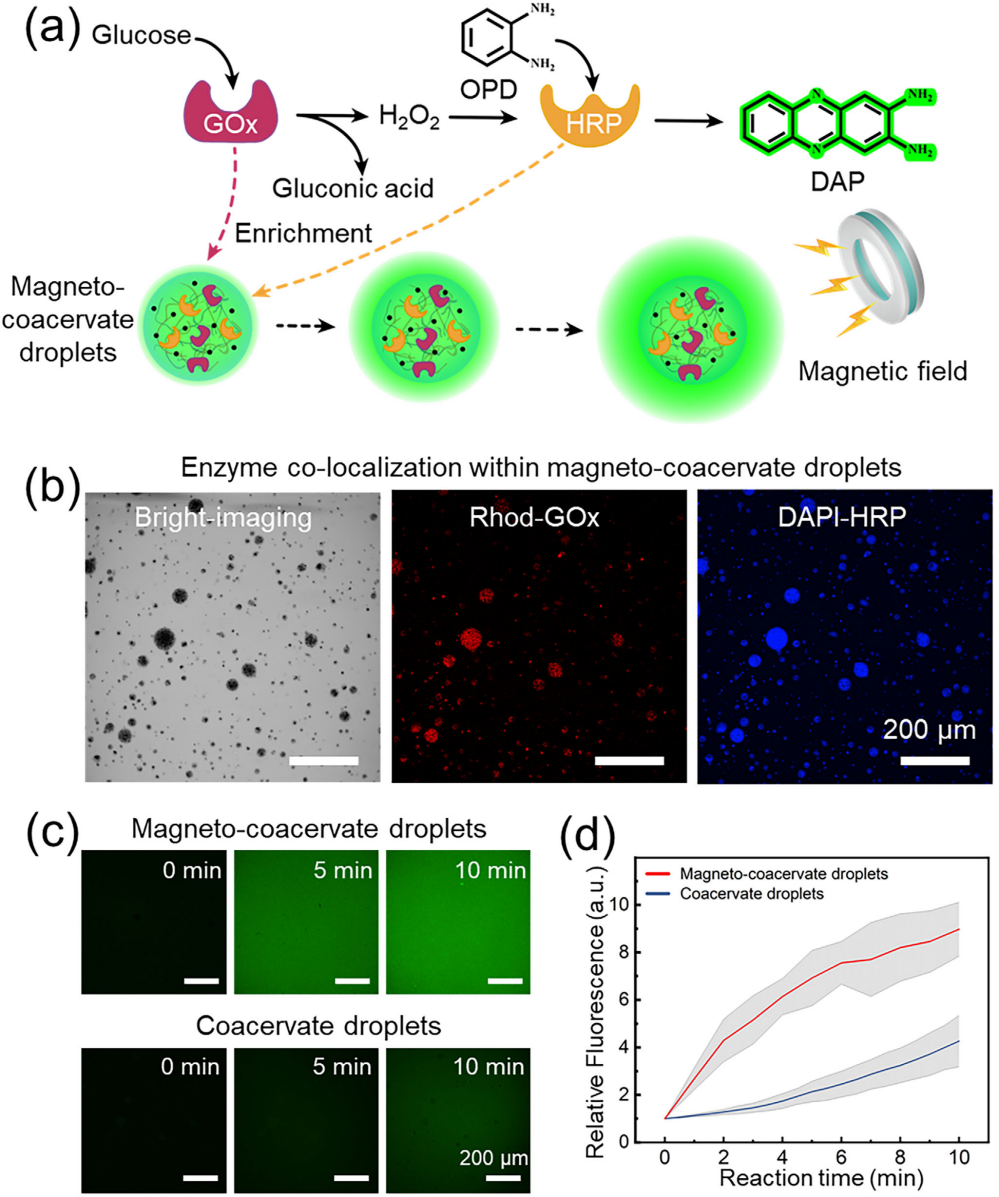
Enhanced catalytic efficiency of enzyme cascade reactions using magneto‐coacervate droplets. (a) Schematic illustrating the glucose oxidase–horseradish peroxidase (GOx–HRP) enzyme cascade reaction conducted within magneto‐coacervate droplets. (b) Fluorescence microscopy images confirming simultaneous enrichment and co‐localization of GOx (red fluorescence, Rhod‐labeled) and HRP (blue fluorescence, DAPI‐labeled) within magneto‐coacervate droplets, demonstrating their effective role as stable, confined microreactors for enzymatic cascade reactions. Scale bar = 200 µm. (c) Green fluorescence intensity (DAP) demonstrating the product of enzymatic cascade reactions within actively rotating magneto‐coacervate droplets under magnetic field actuation (stirring speed: 200 rpm; top images) compared to static coacervate droplets (bottom row images) at different time intervals. Scale bars = 200 µm. (d) Quantitative fluorescence intensity curves comparing magnetically actuated and static groups, obtained from fluorescence microscopy analysis. Shaded regions indicate standard deviation from triplicate measurements (*n* = 3).

The intrinsic magnetic responsiveness of magneto‐coacervate droplets not only enables precise physical manipulation but also significantly enhances biochemical cascade reactions through selective enzyme enrichment and active mixing. To illustrate this capability, magneto‐coacervate droplets were employed as microreactors to conduct a glucose oxidase (GOx) and horseradish peroxidase (HRP) enzyme cascade reaction, as illustrated in Figure 5a. Specifically, GOx catalyzes the oxidation of glucose into gluconic acid, simultaneously producing hydrogen peroxide (H_2_O_2_). This intermediate H_2_O_2_ is subsequently utilized by HRP to oxidize o‐phenylenediamine (OPD) into the fluorescent product 2,3‐diaminophenazine (DAP), enabling reaction monitoring by fluorescence microscopy. Fluorescence microscopy confirmed the simultaneous enrichment and clear co‐localization of both enzymes inside the magneto‐coacervate droplets, as shown in Figure 5b. Specifically, GOx and HRP, labeled respectively with red (Rhod‐GOx) and blue (DAPI‐HRP) fluorescent markers, were visibly concentrated within individual droplets. The electrostatic enrichment of biomolecules by magneto‐coacervate droplets relies on their tunable surface charge properties; specifically, the negatively charged enzymes, glucose oxidase (GOx, pI 4.2) and horseradish peroxidase (HRP, pI 5.1), are effectively enriched within positively charged magneto‐coacervates due to electrostatic attraction. In contrast, the substrate (o‐phenylenediamine, OPD) and its oxidation product (2,3‐diaminophenazine, DAP) are small neutral molecules, which readily diffuse across the droplet boundary, resulting in a uniform distribution rather than specific enrichment inside the droplets. This enzyme confinement within the droplets significantly increases the local concentrations of enzymes and substrates, providing a favorable microenvironment for enhancing reaction efficiency. Furthermore, active magnetic manipulation markedly accelerated the enzymatic cascade reaction. Under an externally applied rotating magnetic field (20 mT, 3.33 Hz), magneto‐coacervate droplets exhibited dynamic motion, promoting effective mixing and mass transport between droplets and the surrounding medium. Fluorescence microscopy demonstrated rapid and intensified formation of the fluorescent product (DAP) in magnetically driven droplets compared to static droplets without magnetic actuation (Figure 5c). Quantitative fluorescence analysis further highlighted that magnetically actuated magneto‐coacervate droplets achieved significantly higher catalytic efficiency compared to their static counterparts, validating the effectiveness of magnetic stirring in enhancing enzymatic cascade reactions (Figure 5d). These results underscore the potential of magneto‐coacervate droplets as powerful microreactors, capable of significantly enhancing biochemical processes through magnetically controlled enzyme enrichment and active mixing.

**FIGURE 6:**
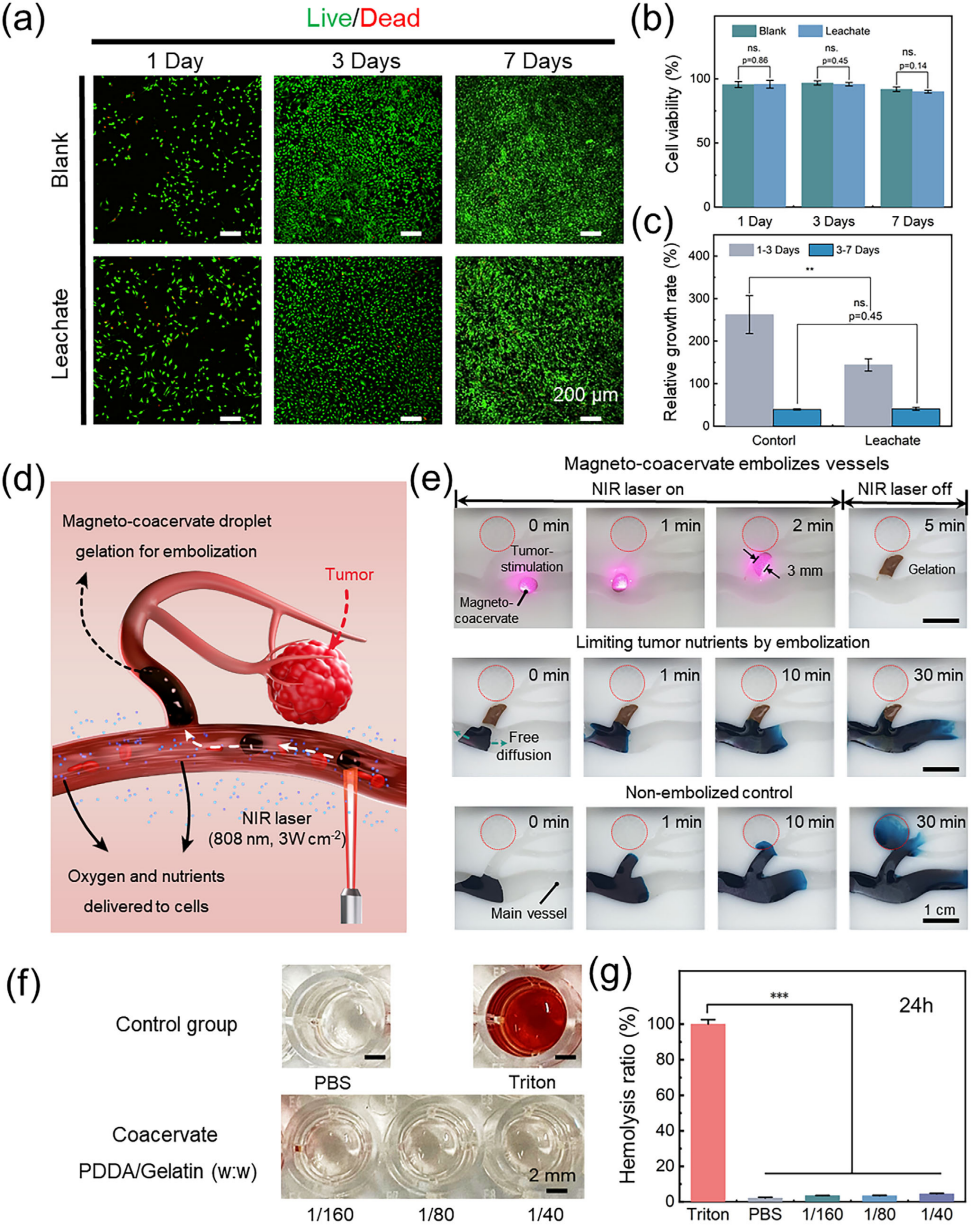
Cytotoxicity evaluation and tumor embolization potential of magneto‐coacervate droplets. (a) Confocal microscopy images demonstrating comparable NIH‐3T3 cell viability at 1, 3, and 7 days between the experimental group (treated with magneto‐coacervate droplet leachate) and the control group (basal medium). Scale bars = 200 µm. (b) Quantitative cell viability assays further confirming no significant differences in cell survival between magneto‐coacervate droplet extracts and the basal medium control. (c) Analysis of cell proliferation rates indicating that magneto‐coacervate droplet leachates do not inhibit NIH‐3T3 cell growth. (d) Schematic illustration depicting the principle of targeted tumor embolization using magneto‐coacervate droplets, where droplets transition into gelled embolic plugs in situ, effectively blocking blood vessels and cutting off nutrient supply to tumor tissue. (e) In vitro embolization experiments demonstrating successful targeted magnetic steering and gelation of magneto‐coacervate droplets at specific vascular sites, resulting in stable vessel occlusion and restricted nutrient diffusion. Control experiments using malachite green dye without droplets show rapid and unrestricted diffusion throughout the vascular model. Scale bars = 1 cm. (f) In vitro hemolysis assay of gelatin/PDDA coacervates. The PBS negative control showed no hemolysis, while the Triton positive control exhibited complete hemolysis (dark red coloration). Gelatin/PDDA coacervate groups (*φ* = 1/160, 1/80, 1/40) displayed results comparable to the PBS group. (g) Quantitative analysis of hemolysis ratio. All error bars represent standard deviation (*n* = 3 independent samples); statistical significance was analyzed via one‐way ANOVA (**p < 0.01, ***p < 0.001).

To confirm the suitability of magneto‐coacervate droplets for biomedical applications, we evaluated their biocompatibility through cytotoxicity assays using NIH‐3T3 cells cultured in droplet extract medium compared to standard culture medium. Confocal microscopy images acquired after 1, 3, and 7 days showed no significant differences in cell viability between cells cultured with droplet extracts and the basal medium control group, verifying the excellent biocompatibility of magneto‐coacervate droplets, as shown in Figure 6a. Quantitative cell viability measurements further supported these findings, indicating consistently high cell viability across all evaluated time points without significant differences from the control group, as shown in Figure 6b. Moreover, analysis of relative cell growth rates revealed no inhibition of cell proliferation due to droplet extracts, reinforcing the biocompatible nature of magneto‐coacervate droplets (Figure 6c). In addition to biocompatibility, magneto‐coacervate droplets demonstrated notable potential in biomedical applications by enabling targeted vascular embolization via magnetically guided in situ gelation, as illustrated in Figure 6d. Using an in vitro embolization model, to enhance the in vivo applicability of this system, we utilized the near‐infrared (NIR) photothermal effect of iron oxide nanoparticles in the droplets to achieve heating. Magneto‐coacervate droplets containing 10 wt% Fe_3_O_4_@SiO_2_ nanoparticles exhibited a heating efficiency of 3°C/s under NIR irradiation at 3 W/cm^2^ (Figure S9). The droplets remained in a liquid state during continuous NIR exposure and were magnetically guided into targeted vascular branches. Upon removal of the NIR stimulus, rapid cooling below the designed gelation temperature induced instantaneous gelation, effectively forming stable embolic plugs. In contrast, the control group treated with a solution of diluted malachite green dye (0.1 wt%) rapidly diffused throughout the simulated vascular network, completely infiltrating the “tumor” region within 10 min. Remarkably, magneto‐coacervate droplets maintained stable vessel occlusion beyond 30 min, effectively blocking nutrient supply and potentially starving tumor tissue. These results highlight the considerable promise of magneto‐coacervate droplets as responsive embolic agents for targeted tumor therapies, demonstrating their capacity for precise magnetic guidance and localized gelation in therapeutic embolization scenarios. We evaluated the hemocompatibility of gelatin/PDDA coacervate droplets with a hemolysis assay. Droplets with different compositional ratios (*φ* = 1/160, 1/80, 1/40; C_PEG_ = 3 wt%) were incubated with diluted erythrocyte suspensions derived from porcine whole blood at 37°C for 24 h. The supernatants obtained after centrifugation were transparent, closely matching the negative control group (PBS), and distinctly different from the positive control (0.1% Triton X‐100). Statistical analysis revealed that the experimental groups significantly differed from the positive control (***p < 0.001), with hemolysis rates consistently below 5%, confirming excellent hemocompatibility.

## 3 Conclusion

In this work, we introduced magneto‐coacervate droplets, a magnetically responsive droplet system created by incorporating Fe_3_O_4_@SiO_2_ nanoparticles into gelatin/PDDA‐based coacervates. This integration successfully overcomes challenges associated with embedding magnetic nanoparticles into fully aqueous coacervate environments, providing robust magnetic responsiveness while preserving the droplets' fluid‐like interiors and intrinsic phase‐separation behaviors. The resulting magneto‐coacervate droplets exhibit dynamic, reversible responsiveness to multiple environmental stimuli, including pH, temperature, and ionic strength, as well as tunable viscosities and sol–gel transitions. Extensive biocompatibility assessments confirm their suitability for biomedical and environmental applications.

Leveraging their unique superparamagnetic properties, magneto‐coacervate droplets demonstrated precise and programmable external manipulation capabilities, including controlled translation, rotation, deformation, splitting, and reconfiguration within complex fluid environments. These functionalities were showcased in diverse application scenarios, which include microplastic capture and recycling and biochemical catalysis enhancement, as well as targeted vascular embolization. The magneto‐coacervate droplets represent a versatile, actively tunable platform that significantly expands the capabilities of conventional coacervate systems. By integrating magnetic responsiveness, selective molecular enrichment, and controllable phase transitions into a single multifunctional droplet design, this technology opens promising avenues in targeted biomedical delivery, adaptive catalytic systems, and sustainable environmental remediation. Future research focusing on optimizing droplet formulations, refining magnetic manipulation techniques, and further exploring their biomedical capabilities will facilitate even broader and more impactful applications.

## 4 Materials and Methods

### 4.1 Preparation of Magneto‐Coacervate Droplets and Phase Diagram Construction

Magneto‐coacervate droplets were prepared by combining gelatin (bloom strength of 250 grams, Aladdin Biochemical Technology Co., China; 10 wt% stock solution), poly(diallyldimethylammonium chloride) (PDDA, Mw <100 000, 35 wt% aqueous solution, Aladdin; diluted to 10 wt% stock solution), polyethylene glycol (PEG, average Mn 8000, Aladdin; 10 wt% stock solution), and deionized water at predetermined ratios to a total volume of 2 mL. Component ratios were systematically adjusted to induce LLPS under controlled conditions (25°C, pH 7.0). After vortex mixing (6000 rpm, 30 s), turbidity measurements (absorbance at 450 nm) and optical microscopy imaging were employed to determine the LLPS boundary, from which a corresponding phase diagram was constructed.

### 4.2 Characterization of pH‐ and Salt‐Responsive Behavior

Magneto‐coacervate droplets with optimized composition (0.01 wt% PDDA, 4 wt% gelatin, and 3 wt% PEG, pH 7.0) were prepared, and their responsiveness to pH variations (adjusted using hydrochloric acid, HCl, 36%–38% purity, Aladdin, and sodium hydroxide, NaOH, ≥96%, Guangzhou Chemical Reagent Co., China) and salt concentrations (NaCl, Guangzhou Chemical Reagent Co., China; 0–200 mM) were studied. Turbidity was measured at 400 nm using a Tecan Spark multimode plate reader to assess droplet stability.

### 4.3 Preparation of Fe_3_O_4_@SiO_2_ Magnetic Nanoparticles

Fe_3_O_4_ nanoparticles (99.0% purity, 20 nm particle size, Aladdin) were coated with SiO_2_ via the Stöber method. Briefly, 10 g Fe_3_O_4_ nanoparticles were dispersed in 600 mL ethanol solution (75% v/v, Aojiaobao, China) with 30 mL ammonium hydroxide (28%–30% NH_3_ basis, Aladdin) under vigorous stirring. Subsequently, 1.5 mL tetraethyl orthosilicate (TEOS, 98%, Aladdin) was gradually added and reacted for 12 h under continuous stirring. Resulting Fe_3_O_4_@SiO_2_ nanoparticles were washed 4–5 times with deionized water, then dried at 60°C for 6 h prior to use.

### 4.4 Fluorescence Recovery After Photobleaching (FRAP) Analysis

Temperature‐induced sol–gel transitions of magneto‐coacervate droplets were characterized by FRAP experiments using confocal laser scanning microscopy (Nikon Ti2‐E) with a 20× objective. Droplets labeled with the fluorescent AIEgen probe TTVP (provided by Xi'an Qiyue Biotechnology Co., Ltd., China) were photobleached using a 488 nm laser. Fluorescence recovery within the bleached region was recorded continuously for 3 min, quantified, and normalized using ImageJ software.

### 4.5 Rheological Characterization

The rheological behavior of magneto‐coacervate droplets was analyzed using a rotational rheometer (DHR‐3, TA Instruments, USA). A 20 mm parallel‐plate geometry (gap: 200 µm) was used at a constant strain (10%) and frequency (10 rad/s). Storage modulus (*G*′), loss modulus (*G*″), and viscosity were measured as functions of temperature. Data analysis was performed using TA Instruments TRIOS software.

### 4.6 Design and Operation of Magnetic Manipulation Platform

A custom magnetic control platform consisting of six orthogonally arranged electromagnets was developed. Each electromagnet provided a surface magnetic field of ∼60 mT with gradients up to 8 mT/mm, enabling precise 3D manipulation of magneto‐coacervate droplets. Joystick‐generated signals (Xbox controller, Microsoft) were converted and amplified to precisely control electromagnet currents, thus facilitating real‐time gradient field modulation and droplet movement with submillimeter precision.

### 4.7 Motion Performance Analysis of Magneto‐Coacervate Droplets

Motion performance of magneto‐coacervate droplets containing 10 wt% Fe_3_O_4_@SiO_2_ was evaluated in aqueous solutions of varying viscosities adjusted by polyethylene glycol (PEG, average Mn 8000, Aladdin) at 25°C. Droplet motion was recorded at 240 fps using an iPhone 11 Pro Max camera. Trajectories and velocities were quantitatively analyzed using MATLAB software to investigate the effects of fluid viscosity on droplet mobility.

### 4.8 Magneto‐Coacervate Droplet Microreactors for Enzyme Cascade Reactions

Magneto‐coacervate droplets encapsulating glucose oxidase (GOx, ≥10 000 units/g, Merck Sigma–Aldrich, USA; 0.05 U/mL) and horseradish peroxidase (HRP, ≥250 units/mg, Merck Sigma–Aldrich, USA; 0.05 U/mL) were utilized as enzyme microreactors. Glucose (0.2 mmol/L) and o‐phenylenediamine (o‐PD, Merck Sigma–Aldrich, USA; 0.4 mmol/L) served as substrates. In static experiments, fluorescence of the reaction product, 2,3‐diaminophenazine (DAP), was measured (excitation/emission: 433/560 nm) every 5 min using a TECAN Spark plate reader. For magnetically actuated experiments (droplets with 5 wt% Fe_3_O_4_@SiO_2_), a rotating magnetic field (200 rpm) was applied, and reaction progression was monitored using confocal microscopy. Experiments were conducted in triplicate.

### 4.9 Rotating Magnetic Field Programming

A custom algorithm generated real‐time rotating magnetic fields based on input angular velocity and direction. Magnetic field updates occurred at 2400 Hz, ensuring continuous rotation and precise droplet manipulation. At 200 rpm (1200°/s), magnetic field updates corresponded to ∼0.5° per step, fully satisfying system resolution requirements.

### 4.10 Cytotoxicity Assessment

Cytotoxicity of magneto‐coacervate droplets was assessed using NIH‐3T3 cells. Magneto‐coacervate droplets (0.0105 wt% PDDA, 4 wt% gelatin, and 3 wt% PEG) were gelled, immersed in high‐glucose DMEM (Gibco, USA) containing 10% fetal bovine serum (FBS, Gibco, USA) and 1% penicillin–streptomycin (Gibco, USA) at 25°C for 24 h, and filtered through 0.22 µm membranes. NIH‐3T3 cells (3 × 10^4^ cells/well) were cultured with droplet extracts for 7 days (37°C, 5% CO_2_). Cell viability was determined on days 1, 3, and 7 by acridine orange (AO)/propidium iodide (PI) double staining (Solarbio, China), with fluorescence images analyzed using ImageJ software.

### 4.11 Hemolysis Activity Assay

Porcine whole blood was centrifuged (200 × g, 10 min) to isolate erythrocytes, which were subsequently washed three times with phosphate‐buffered saline (PBS, pH 7.4) and diluted to 5% (v/v) in PBS. Gelatin/PDDA coacervate droplets (20 mg) with varying component ratios (*φ* = 1/160, 1/80, 1/40; C_PEG_ = 3 wt%) were individually added to 500 µL of the erythrocyte suspension, followed by incubation at 37°C for 24 h. Negative control (100 µL normal saline) and positive control (0.1% Triton X‐100) were established in parallel. Post‐incubation, the suspensions were centrifuged (500 × g, 15 min), and supernatants were transferred to a 96‐well clear plate. Absorbance at 540 nm was measured using a microplate reader (Thermo Scientific Multiskan GO). The hemolysis ratio was calculated as:

$$\begin{array}{ccc} \mathrm{Hemolysis}\;\left(\%\right) & = & \left(\mathrm{OD}\;\mathrm{of}\;\mathrm{sample}-\mathrm{OD}\;\mathrm{of}\;\mathrm{fresh}\;\mathrm{PBS}\right)/\\ & & \left(\mathrm{OD}\;\mathrm{of}\;\mathrm{positive}-\mathrm{OD}\;\mathrm{of}\;\mathrm{fresh}\;\mathrm{PBS}\right)\times 100. \end{array}$$

### 4.12 Zeta Potential Measurements

Zeta potentials of magneto‐coacervate droplets prepared in deionized water (pH 7.0) were measured by dynamic light scattering (Zetasizer Nano ZS, Malvern Panalytical Ltd, UK) at 25°C. Measurements were conducted in triplicate, with results expressed as mean ± standard deviation to investigate surface charge modulation resulting from compositional variations.

## Author Contributions

Z.L., C.Q., and T.T.K. conceived, designed, and supervised the project. K.L. performed experiments. Z.L., C.Q., and T.T.K. analyzed the data. Z.L. and T.T.K. wrote the manuscript. The manuscript was written through the contributions of all authors. All authors have given approval to the final version of the manuscript.

## Conflicts of Interest

The authors declare no conflicts of interest.

## Supporting information




**Supporting File**: advs76398‐sup‐0001‐SuppMat.docx.

## Data Availability

The data that support the findings of this study are available from the corresponding author upon reasonable request.
