# Supplementary material for: Multi‐Stimuli Responsive Magneto‐Coacervate Droplets for Selective Molecular Enrichment and Programmable Manipulation
Source: Adv Sci (Weinh). 2026 Jul 11;13(54):e76398. doi: 10.1002/advs.76398 (PMC13355930; doi:10.1002/advs.76398)
Supplement: Supplementary file 1 [file ADVS-13-e76398-s001.docx]

**Supplementary Material**

**Multi-Stimuli Responsive Magneto-Coacervate Droplets for Selective Molecular Enrichment and Programmable Manipulation**

*Kailang Liu^1,2^, Haocheng Ran^3^, Haohui Ou^3^, Peiying Chen^3^, Cheng Qi^2^, Tiantian Kong^3,4^, Zhou Liu^1,*^*

^1^College of Chemistry and Environmental Engineering, Shenzhen University, Shenzhen, Guangdong 518000, China

^2^College of Mechatronics and Control Engineering, Shenzhen University, Shenzhen, Guangdong 518000, China

^3^Department of Biomedical Engineering, School of Medicine, Shenzhen University, Shenzhen, Guangdong 518000, China

^4^Department of Urology, Shenzhen Institute of Translational Medicine, The First Affiliated Hospital of Shenzhen University, Shenzhen Second People’s Hospital, Shenzhen, Guangdong 518037, China

* zhouliu@szu.edu.cn (Z. Liu)

**Keywords:** coacervate, condensate, droplet, magnetic actuation

**Contents:**

(1) Figure S1. TEM-EDS characterization of Fe₃O₄@SiO₂ magnetic nanoparticles.
(2) Figure S2. SEM-EDS characterization of magneto-coacervate droplets loaded with Fe₃O₄@SiO₂ nanoparticles.
(3) Figure S3. FT-IR spectra comparison between Fe₃O₄@SiO₂ nanoparticles and magneto-coacervate droplets.
(4) Figure S4. Long-term stability of Fe₃O₄@SiO₂ magnetic nanoparticles loaded within Gelatin/PDDA coacervate droplets.
(5) Figure S5. Gelatin/PDDA coacervate phase behavior under different salt conditions.
(6) Figure S6. Magnetic hysteresis analysis: magneto-coacervate droplet vs. magneto-gel.
(7) Figure S7. Axial magnetic field distribution characteristics of a single electromagnetic coil.
(8) Figure S8. Deformation and splitting of magneto-coacervate droplet under a magnetic field.
(9) Figure S9. Near-infrared photothermal response characteristics of magneto- coacervate droplet.
(10) Table S1: Correlation between PEG solution viscosity and concentration.

**Figure S1.** **TEM-EDS characterization of Fe₃O₄@SiO₂ magnetic nanoparticles.** (a) TEM image shows nanoparticles with a clear core-shell structure, ranging from approximately 10 to 30 nm in diameter. (b-e) Corresponding EDS elemental mappings confirm the uniform distribution of Fe, Si, and O elements, validating the presence and uniform coating of the SiO₂ shell on the Fe₃O₄ core.

**Figure S2. SEM-EDS characterization of magneto-coacervate droplets loaded with Fe₃O₄@SiO₂ nanoparticles.** (a) SEM image displaying the porous spherical structure of an individual freeze-dried magneto-coacervate droplet; (b)-(e) Corresponding EDS elemental mappings clearly demonstrate the distribution of Fe (yellow), N (orange), Si (cyan), and O (green), confirming the successful incorporation of Fe₃O₄@SiO₂ nanoparticles within the droplet matrix.

**Figure S3. FT-IR spectra comparison between Fe₃O₄@SiO₂ nanoparticles and magneto-coacervate droplets.**

**Figure S4. Long-term stability of Fe₃O₄@SiO₂ magnetic nanoparticles loaded within Gelatin/PDDA coacervate droplets.** Bright-field time-lapse images of magneto- coacervate droplets(*φ* = 1:31, C_PEG_ = 3 wt%) containing 10 wt% Fe₃O₄@SiO₂ nanoparticles.

**Figure S5. Salt-induced phase behavior of magneto-coacervate droplets.** The turbidity of magneto-coacervate droplets prepared with varying PDDA-to-gelatin ratios (φ = 1:63, 1:31, and 1:15, with fixed PEG at 3 wt%) decreases progressively with increasing NaCl concentration (0–200 mM). Increased ionic strength enhances electrostatic shielding, progressively disrupting droplet structure and ultimately causing complete dissolution. Turbidity measurements were conducted using a microplate reader at 400 nm. Data represent mean ± SD (n = 3 independent experiments).

**Figure S6. Magnetic hysteresis analysis of magneto-coacervate droplets in liquid and gel states.** Characteristic hysteresis loops of magneto-coacervate droplets in liquid (blue curve) and gel (red curve) states demonstrate soft magnetic behavior (coercivity H_c_ < 100 Oe). Compared to the droplet state (Ms = 2.0 emu/g), the gel state exhibits approximately 32% lower saturation magnetization (Ms = 1.5 emu/g) and a wider hysteresis loop, indicating reduced magnetic responsiveness. This reduction is attributed to the restricted mobility of magnetic nanoparticles within the crosslinked gel network. Both states achieve complete magnetization reversal below 20 kOe, satisfying criteria for soft magnetic materials.

**Figure S7. Axial magnetic field distribution of a single electromagnetic coil. (a)** Variation of magnetic flux density along the axial distance at an applied coil voltage of 39 V. The red solid line shows an exponential fit (y = a exp(-x/b) + c, adjusted R² = 0.9962). **(b)** Linear relationship between magnetic flux density and applied voltage measured at the coil core surface (axial distance = 0 mm). The red solid line indicates a linear fit (y = a + bx, adjusted R² = 0.9921).

**Figure S8. Magnetically induced deformation and splitting of a magneto-coacervate droplet.** Time-sequence microscopic images (from left to right) depict structural reconfiguration of a magneto-coacervate droplet under a bidirectional magnetic field (50 mT): the initially spherical droplet (0 s) elongates into an ellipsoid (8 s), develops a slender fluid bridge (11–16 s), undergoes necking (22 s), and eventually splits (42 s). This behavior demonstrates capillary-driven rupture induced when the magnetic tensile stress exceeds a critical threshold, highlighting the intrinsic viscoelastic and dissipative characteristics of magneto-coacervate droplets. Experimental conditions: 25 °C, magnetic field strength: 50 mT.

**Figure S9. Near-infrared photothermal response characteristics of magneto- coacervate droplet** (10 wt% magnetic particles). (a) Time-lapse thermal images (0 s, 4 s, 13 s, 22 s) demonstrate temperature evolution of the droplet. Scale bar: 5 mm. (E) Quantified thermal parameter exhibits a sustained monotonic increase over time with a heating rate of ~3 °C/s (error bars = ±1 SD, n=3).

| **PEG concentration (wt.%)** | 5 | 10 | 15 | 20 | 25 | 30 |
| --- | --- | --- | --- | --- | --- | --- |
| **Dynamic Viscosity of PEG solution (mPa·s)** | 3.52 | 6.90 | 12.30 | 21.52 | 35.88 | 52.11 |

**Table S1. Relationship between PEG concentration and solution viscosity.** Polyethylene glycol (PEG) with an average molecular weight of 8,000 Da was employed. All solutions listed contain sucrose at a fixed concentration of 0.2 g/mL. The inclusion of sucrose intentionally increases the density difference between the solution and magneto-coacervate droplets, promoting droplet flotation at the solution surface. This density adjustment significantly enhances visualization and facilitates clear tracking of magneto-coacervate droplet movement.
